# Stereoselective ketamine effect on cardiac output: a population pharmacokinetic/pharmacodynamic modelling study in healthy volunteers

**DOI:** 10.1016/j.bja.2021.02.034

**Published:** 2021-04-22

**Authors:** Jasper Kamp, Monique van Velzen, Leon Aarts, Marieke Niesters, Albert Dahan, Erik Olofsen

**Affiliations:** Department of Anesthesiology, Leiden University Medical Center, Leiden, the Netherlands

**Keywords:** ketamine, cardiac output, stereoselectivity, modeling, NONMEM, norketamine, hydroxynorketamine, metabolomics

## Abstract

**Background:**

Ketamine has cardiac excitatory side-effects. Currently, data on the effects of ketamine and metabolite concentrations on cardiac output are scarce. We therefore developed a pharmacodynamic model derived from data from a randomised clinical trial. The current study is part of a larger clinical study evaluating the potential mitigating effect of sodium nitroprusside on the psychedelic effects of ketamine.

**Methods:**

Twenty healthy male subjects received escalating esketamine and racemic ketamine doses in combination with either placebo or sodium nitroprusside on four visits: (i) esketamine and placebo, (ii) esketamine and sodium nitroprusside, (iii) racemic ketamine and placebo, and (iv) racemic ketamine and sodium nitroprusside. During each visit, arterial blood samples were obtained and cardiac output was measured. Nonlinear mixed-effect modelling was used to analyse the cardiac output time-series data. Ketamine metabolites were added to the model in a sequential manner to evaluate the effects of metabolites.

**Results:**

A model including an *S*-ketamine and *S*-norketamine effect best described the data. Ketamine increased cardiac output, whereas modelling revealed that *S*-norketamine decreased cardiac output. No significant effects were detected for *R*-ketamine, metabolites other than *S*-norketamine, or sodium nitroprusside on cardiac output.

**Conclusions:**

*S*-Ketamine, but not *R*-ketamine, increased cardiac output in a dose-dependent manner. In contrast to *S*-ketamine, its metabolite *S*-norketamine reduced cardiac excitation in a dose-dependent manner.

**Clinical trial registration:**

Dutch Cochrane Center 5359.

**Editor's key points**

• Although ketamine has a direct negative inotropic effect, it generally increases heart rate and blood pressure by stimulating the sympathetic system.
• The influence of the isomers of ketamine and their metabolites on cardiac output has not been studied in detail.
• The authors analysed data from a previously performed study during which cardiac output and the concentrations of the isomers and their metabolites were measured.
• *S*-Ketamine increased cardiac output, but *S*-norketamine reduced cardiac excitation, whereas *R*-ketamine and its metabolites had no effect on cardiac output.

Ketamine exhibits a plethora of significant adverse effects, including those on the cardiovascular system.^1^ Although ketamine has a direct negative inotropic effect, activation of the sympathetic system causes release of catecholamines, vagal inhibition, norepinephrine release from sympathetic ganglia neurones, and inhibition of norepinephrine reuptake at neuronal and non-neuronal tissue (including the myocardium).^2^, ^3^, ^4^ As a consequence, ketamine induces cardiac depression when norepinephrine stores are depleted. If stores are not depleted, anaesthetic doses of ketamine induce cardiac excitation (often a short period of cardiac depression precedes excitation) as do low or sub-anaesthetic doses of ketamine, such as those used in the treatment of acute and chronic pain. Cardiovascular excitation is characterised by systemic and pulmonary hypertension, tachycardia, and increases in cardiac output, all combined with an increase in myocardial oxygen consumption. Cardiac depression may be partially explained by a decrease in intracellular Ca^2+^ levels as a result of the ketamine-induced inhibition of Ca^2+^ release from intracellular stores and inhibition of the L-type voltage-gated Ca^2+^ channels.^5^^,^^6^ The exact mechanism of ketamine-induced sympathetic activation is not known, but may be related to Na^+^ channel blockade in parasympathetic centres in the brainstem and in spinal cord neurones.^7^ Additionally, reduction of intracellular nitric oxide concentrations has been proposed as a mechanism of sympathetic activation.^8^

In the current study, we examined the effects of racemic (containing both *R*- and *S*-ketamine) and separately *S*-ketamine and their most relevant metabolites norketamine (NK), dehydronorketamine (DHNK), and hydroxynorketamine (HNK) on cardiac output in a population of healthy volunteers. We analysed the data using a population pharmacokinetic/pharmacodynamic modelling approach to separate the effects of *S*- and *R*-ketamine (and metabolites) on cardiac output. This study is part of a larger project, in which the effects of nitric oxide donor sodium nitroprusside (SNP) on racemic (*RS*)- and *S*-ketamine-related adverse effects was studied. We previously reported that SNP reduces ketamine-induced schizotypical adverse effects after *RS*-ketamine, but not after *S*-ketamine, suggestive of an SNP effect on a pathway activated by the *R*-ketamine isomer.^9^ More recently, we published a pharmacokinetic model of ketamine and its metabolites, and concluded that the SNP effects were not induced by changes in ketamine pharmacokinetics.^10^ Our current analysis is aimed at determining the separate effects of *S*- and *R*-ketamine isomers and related metabolites on cardiac output, and determining whether SNP has a mitigating effect on ketamine-induced cardiovascular excitatory effects.

## Methods

### Ethics and subjects

This study is part of a large project on the ability of SNP to reduce *RS*- and *S*-ketamine-induced side-effects. Apart from the primary analysis,^9^ three separate secondary analyses were pre-planned: (i) development of a population pharmacokinetic model of *RS*- and *S*-ketamine and metabolites^10^; (ii) development of a pharmacodynamic model of the analgesic and schizotypical side-effects of *RS*- and *S*-ketamine; and finally, (iii) development of a population pharmacodynamic model that describes the changes induced by *RS*- and *S*-ketamine on cardiac output and effect of SNP. Here, we report the results of the last analysis. The medical ethic committees of the Leiden University Medical Center (Medisch Ethische Toetsingscommissie Leiden, Den Haag, Delft, the Netherlands) approved the study protocol that was registered at the trial registry of the Dutch Cochrane Center (https://www.trialregister.nl/) under registration number 5359. All study procedures followed the latest version of the Good Clinical Practice guidelines and the Declaration of Helsinki. The subject selection process can be found in Jonkman and colleagues.^9^ In brief, inclusion criteria were healthy male participants, aged 18–35 yr, and BMI of 19–30 kg m^−2^. They were all screened, and only after their history and physical examination (including negative drug tests) did not yield any abnormalities, the subjects were enrolled in the study. Participants were not allowed to consume caffeinated food or drinks, or consume any grapefruit-containing products in the day and week, respectively, before dosing.

### Study design

The study had a double-blind, randomised, four-way crossover design. All subjects received escalating i.v. doses of *RS*-ketamine (Ketalar®; Pfizer Pharma, Berlin, Germany) on visits A and B, and escalating doses of *S*-ketamine (Ketanest®; Eurocept B.V., Ankeveen, the Netherlands) on visits C and D. On visits A and C, SNP was infused at a dose of 0.5 mg kg^−1^ min^−1^, whereas placebo (NaCl 0.9%) was infused on visits B and D. *RS*-/*S*-ketamine and SNP/placebo were administered via two separate infusion lines placed on opposing arms. The order of visits was randomised using a computer-generated randomisation list based on a four-block design (www.randomization.com). Blinding procedures, allocation, and dispensing are described elsewhere. The researchers were unblinded after all experiments were concluded (August 24, 2017).

*RS*-ketamine and *S*-ketamine were dosed as follows: *RS*-ketamine 60 min: 0.28 mg kg^−1^ h^−1^; 60–120 min: 0.57 mg kg^−1^ h^−1^, and 120–180 min: 1.14 mg kg^−1^ h^−1^; *S*-ketamine 0–60 min: 0.14 mg kg^−1^ h^−1^; 60–120 min: 0.28 mg kg^−1^ h^−1^, and 120–180 min: 0.57 mg kg^−1^ h^−1^. These doses were considered equipotent in terms of analgesic effect.^9^ Arterial blood samples were obtained from an arterial line at the following times relative to the start of drug infusion (*t*=0): *t*=2, 6, 30, 59, 62, 66, 100, 119, 122, 126, 150, 179, 182, 186, 195, 210, and 300 min. Plasma samples were analysed in the laboratory of Dr Evan Kharasch as described by Rao and colleagues.^11^ After *RS*-ketamine administration, the plasma concentration of *S*- and *R*-ketamine, *S*- and *R*–NK, and *S*- and *R*-DHNK, and total (*S*+*R*) HNK were measured. Cardiac output was measured from the arterial pressure wave (obtained from the arterial cannula) using the FloTrac® sensor and Vigileo monitor (Edwards Lifesciences, USA) Cardiac-output values were averaged over 1 min intervals for further analysis.

### Population pharmacokinetic/pharmacodynamic analysis

NONMEM version 7.4.4 (ICON Development Solution, Hanover, MD, USA) was used for the data analyses. The plasma concentration/cardiac-output data were analysed by a two-step pharmacokinetic/pharmacodynamic approach. Firstly, a pharmacokinetic model was developed as described previously.^10^ In brief, a seven-compartment pharmacokinetic model was constructed to describe the pharmacokinetics of ketamine, NK, DHNK enantiomers, and total HNK. The central compartment of a two-compartmental ketamine model was linked via two metabolic (or delay) compartments to the central compartment of a two-compartmental NK model. As NK is further metabolised to either DHNK or HNK, the central NK compartment was linked to the DHNK disposition compartment via one metabolic (or delay) compartment; HNK was modelled with a two-compartmental model, of which the central compartment was linked to the central NK compartment without a delay compartment. See also Fig. 2 of Kamp and colleagues.^10^

In the second step, the empirical Bayesian estimates obtained from the pharmacokinetic analysis were used as input for the (cardiac output) pharmacodynamic model. Random effects were included in the model to account for inter-individual variability and inter-occasion variability (IOV), as follows: θ_*i*_=θ × exp (η_*i*_+η_iov_), where θ_*i*_ is the parameter for individual *i*, θ is the population parameter, η_*i*_ is the random difference between the population and the individual parameter, and η_iov_ is the difference between θ_*i*_ and θ attributable to IOV.

To test the potential effect of each compound, we started with a base pharmacodynamic model that just included *S*-ketamine, which was sequentially expanded by adding its metabolites, and next *R*-ketamine and its metabolites. The total effect on cardiac output was defined as the sum of effects calculated for each compound. Compounds were only included in the pharmacodynamic model when addition gave a significant (*P*<0.01) improvement of the objective function value, as calculated by NONMEM. To evaluate a potential hysteresis between ketamine and metabolite plasma concentrations and observed effects, postulated effect compartments were tested for each individual included compound (i.e. we tested whether effect equilibration compartments improved the objective function value). It was assumed that the effect compartment equilibrates with the central plasma compartment with rate constant *k*_*e*0_ with effect half-time *t*_1/2_=ln (2)/*k*_*e*0_.

A linear pharmacodynamic model was initially developed to describe the plasma concentration/cardiac-output data (i.e. the base model): YF=BLN ∗ (1+YE_SUM_)+ε, where YF is the cardiac-output value predicted by the model, BLN is the baseline cardiac output, YE_SUM_ is the sum of the effects on the cardiac output caused by ketamine and its metabolites (i.e. YE_SUM_=YE_*X*1_+ … +YE_*X*7_), and ε is the residual error. The individual effect of each compound on cardiac output was defined by YE_Xn_=0.25 · (*C*_Xn_/*C*_25Xn_)^γ^, where YE_Xn_ is the effect of compound Xn on cardiac output, γ is the Hill coefficient, *C*_Xn_ is the drug concentration, and *C*_25Xn_ is the effect-site concentration of compound Xn that leads to a 25% change of cardiac output relative to baseline (25% is in the midst of the observed changes) of compound Xn contributing to changes in total cardiac output, where Xn ranges from *X*1 to *X*7, with *X*1 *S*-ketamine, *X*2 *R*-ketamine, *X*3 *S*-NK, *X*4 *R*–NK, *X*5 *S*-DHNK, *X*6 *R*-DHNK, and *X*7 total HNK. Note that *C*_Xn_ could be either the drug concentration in the central volume of distribution or in the effect compartment, depending on the compound.

As undershoot was observed in the cardiac-output data after termination of ketamine infusion, a control mechanism was added to the model: YF=BLN ∗ (1+YE_SUM_–YC) and τ dYC/dt=(YE_SUM_–YC), where YC is the output of the controller that counteracts YE_SUM_ with time constant τ. In addition, as in some subjects the residuals of the data fits were correlated, a parallel process noise component (i.e. Kalman filter) was added to the model: dYC=(YE_SUM_–YC)/τ · d*t*+σ_*ν*_ · d*w*, where σ_*ν*_ is the standard deviation (sd) of the noise component (with units L min^−1^ min^−0.5^) and d*w* a stochastic (Wiener process), with units for w min^0.5^. Finally, a trend parameter (TRD) was added to the model, because a clear increasing trend, irrespective of ketamine or metabolite concentrations, was observed: YF=BLN ∗ (1+YE_SUM_–YC+TRD ∗ *t*/300), where *t* is the time from the start of the experiment in minutes.

Model selection was based on significant improvements in the objective function value (–2 loglikelihood with *P*<0.01 after a χ^2^ distribution) and by assessment of individual model fits and goodness-of-fit plots (population predicted *vs* observed, individual predicted *vs* observed, conditional weighted residuals *vs* time, and conditional weighted residuals *vs* population predicted plots) and the visual predictive checks. Additionally, auto- and cross-correlation plots were assessed to evaluate model goodness of fit. The correlation between two residuals shifted in time can be described by an auto-correlation function, in which residuals are uncorrelated (so-called white residuals) when the auto-correlation function is equal to zero, with the exception when *t*=0. In addition, the correlation between the residuals and input (i.e. the model output, before being inputted in the Kalman filter) shifted in time can be described by the cross-correlation function. Similar to the auto-correlation function, if the cross-correlation function equals zero, this indicates that the residuals are completely random and the model therefore explains the data completely.^12^

As a large number of combinations could be tested because of the potential effects of seven different compounds and the incorporation of the TRD parameter, controller, and Kalman filter in the model, we here only describe the most important model combinations. Sequential testing with ketamine and metabolites was performed for five models:

(i) Model 1: base model with just the Kalman filter (no trend parameter or controller); note that when YE_SUM_=0, the controller is deactivated
(ii) Model 2: model 1+trend parameter
(iii) Model 3: model 1+controller
(iv) Model 4: model 1+trend parameter+controller
(v) Model 5: model 2 without the Kalman filter

The controller relates the undershoot in cardiac output after ketamine infusion ended, the trend term relates to a slow increase in cardiac output over time, and the Kalman filter to the noise in the data.

Finally, potential covariates were tested on the best model, by an automated stepwise covariate screening algorithm (stepwise covariate model building module from Perl Speaks NONMEM).^13^ Tested covariates were (i) *S*- or *RS*-ketamine administration and (ii) placebo or SNP administration. Firstly, a forward search was performed, adding covariates to the model that caused a significant reduction (*P*<0.01) of the objective function value. Potential covariates were added to the model parameters in a linear relation, described as θ_*i*_=θ_ref_ × (1+θ_COV_), where θ_ref_ is the typical parameter value for a subject belonging to the reference category of the covariate and θ_COV_ is the effect of belonging to the non-reference category. Once covariates caused no further reduction in objective function value, the backward search was started. In this step, covariates were sequentially removed from the model. When removal caused a significant reduction of the objective function value (*P*<0.001), the covariate was retained in the model. This process was continued until all covariates were excluded or until no more covariates were left to exclude. To limit the risk of including irrelevant covariates, the backward search was performed with a more stringent selection criterion.

## Results

**Fig 1:**
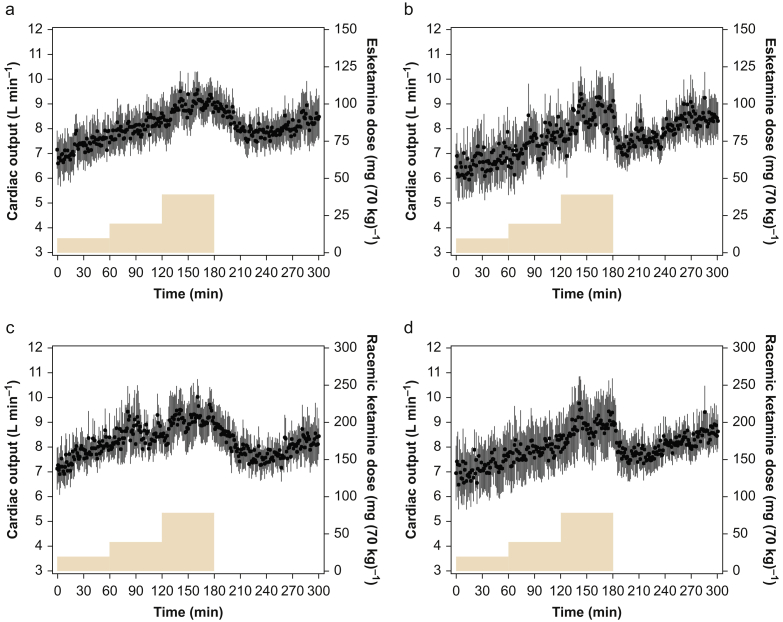
Mean time/cardiac-output curves (a and b) after *S*-ketamine with either placebo or sodium nitroprusside (SNP) co-administration, and (c and d) after *RS*-ketamine with either placebo or SNP co-administration. Data are mean [standard deviation]. Ketamine doses are given in yellow (right y-axis).

All 20 subjects successfully completed the study without serious adverse effects. Mean [sd (range)] subject age was 23 [2 (19–28)] yr, height 186 [6 (175–193)] cm, body weight 83 [9 (60–98)] kg, and BMI 24 [2.1 (19.5–28.4)] kg m^−2^. Cardiac-output data were obtained from all subjects, except from Subject 19. We did not collect cardiac-output data on one occasion because of failure of insertion of the arterial line. Mean cardiac output *vs* time curves are shown in Fig. 1.

**Fig 2:**
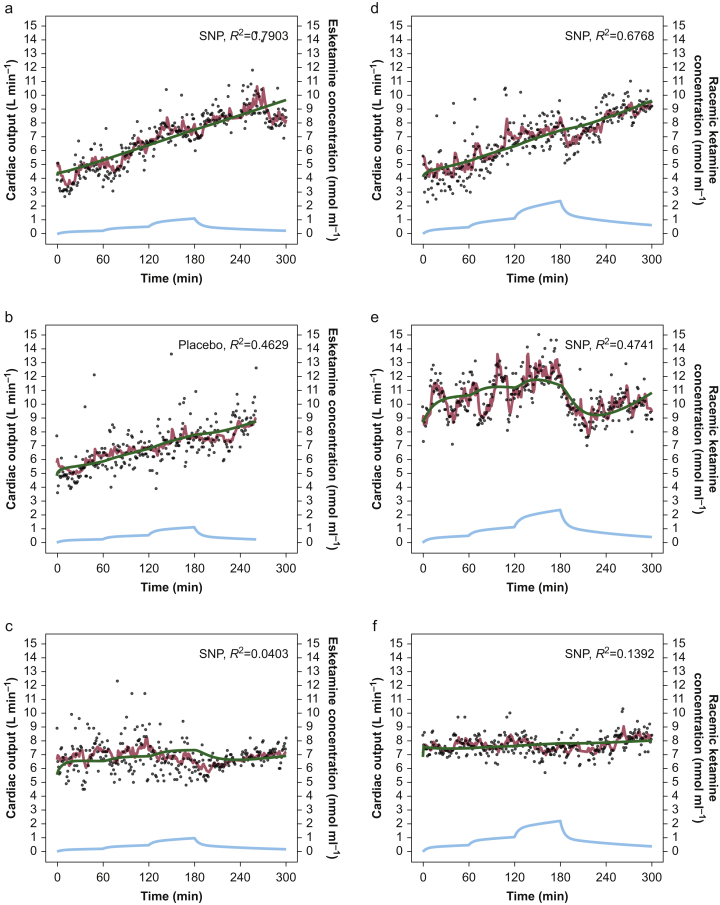
Pharmacodynamic model fits. (a) Best, (b) median, and (c) worst cardiac-output model fits after esketamine administration, and (d) best, (e) median, and (f) worst cardiac-output model fits after racemic ketamine administration. The dots are the measured data, and the red and green lines the output of Models 2 (with Kalman filter) and 5 (without Kalman filter), respectively. The blue lines are the simulated *S*-ketamine concentrations (right y-axis), based on the empirical Bayesian estimates obtained from Kamp and colleagues.^10^.

### Pharmacodynamic models

Starting with Model 1 (base model with Kalman filter; absolute objective function value 24 517), adding the trend term resulted in a ΔOFV of –74 points (Model 2). No significant improvement was observed when the controller was added to Model 1 (Model 3). As the structure of Model 2 best described the data, we limited the description of the sequential compound testing to Model 2. The effect of *S*-ketamine on cardiac output was best modelled by adding an effect compartment (ΔOFV of –9.41 points). Sequential expansion of the model with metabolites only showed a significant effect of *S*-NK (ΔOFV of –18 points), but in contrast to *S*-ketamine, reducing cardiac output. Adding *R*-ketamine or its metabolites did not cause a significant improvement of the model, and these were therefore not incorporated. Finally, adding an *S*-NK effect compartment improved the model (ΔOFV of –11 points). In agreement with these findings, sequential compound testing of Models 1 and 3–5 failed to show significant metabolite effects, indicating that the trend term and controller did not obfuscate potential metabolite effects on cardiac output. Removal of the Kalman filter from the final Model 2 resulted in an increase in objective function value by 5986 points and large ω^2^ values, indicating that the Kalman filter significantly improved the model.

Pharmacodynamic parameters of the final model (Model 2) are given in Table 1, with best, median, and worst data fits in Fig. 2. The *S*-ketamine concentration causing an increase in cardiac output by 25% was 1.68 [0.45] nmol ml^−1^. The *S*-ketamine blood effect-site equilibration half-life (*t*½*k*_*e*0_) was 2.28 [0.64] min, the time constant of the noise component was 31.4 [7.9] min, and the value of the trend term was 0.38 [0.08] L (300 min)^−1^ (i.e. a 380 ml min^−1^ increase in cardiac output over the course of the study). In addition, the *S*-NK concentration causing a 25% reduction of cardiac output was 0.67 [0.22] nmol ml^−1^, with an equilibration half-life of 29.3 [16.4] min.

**Table 1:** Pharmacodynamic parameter estimates of Model 2. γ is a shape parameter; TRD is a trend term; *C*_25_*S*-ketamine is the *S*-ketamine plasma concentration that causes a 25% increase in cardiac output; *C*_25_*S*-norketamine is the *S*-norketamine plasma concentration that causes a 25% decrease in cardiac output; *t*_1/2_*k*_*e*0_ is the plasma effect compartment equilibrium half-life; τ is the time constant of the noise compartment; σ_*ν*_ and σ_ε_ are the standard deviations of the process and measurement noise components, respectively. CV, coefficient of variation; see, standard error of the estimate.

	Typical parameter value (see) [% CV]	Inter-individual variability (%) (see) [% CV]	Inter-occasion variability (%) (see) [% CV]
Baseline cardiac output (L min^−1^)	6.8 (0.2) [3]	11.3 (3.4) [29]	9.7 (1.5) [15]
γ	1 FIXED	—	26.4 (8.7) [33]
Trend term (L min^−2^)	0.384 (0.081) [21]	17.1 (3.4) [20]	—
*C*_25_*S*-ketamine (nmol ml^−1^)	1.68 (0.45) [27]	93.8 (20.6) [22]	—
*C*_25_*S*-norketamine (nmol ml^−1^)	0.673 (0.215) [32]	—	
*S*-ketamine *t*_1/2_*k*_*e*0_ (min)	2.28 (0.64) [28]	—	—
*S*-norketamine *t*_1/2_*k*_*e*0_ (min)	29.3 (16.4) [56]		
τ of the noise component (min)	31.4 (7.9) [25]	—	—
σ_*ν*_ (L min^−1^ min^−0.5^)	0.89 (0.05) [6]	22.9 (2.7) [12]	25.4 (3.6) [14]
σ_ε_ (L min^−1^)	0.037 (0.004) [10]	—	35.9 (4.3) [12]

**Fig 3:**
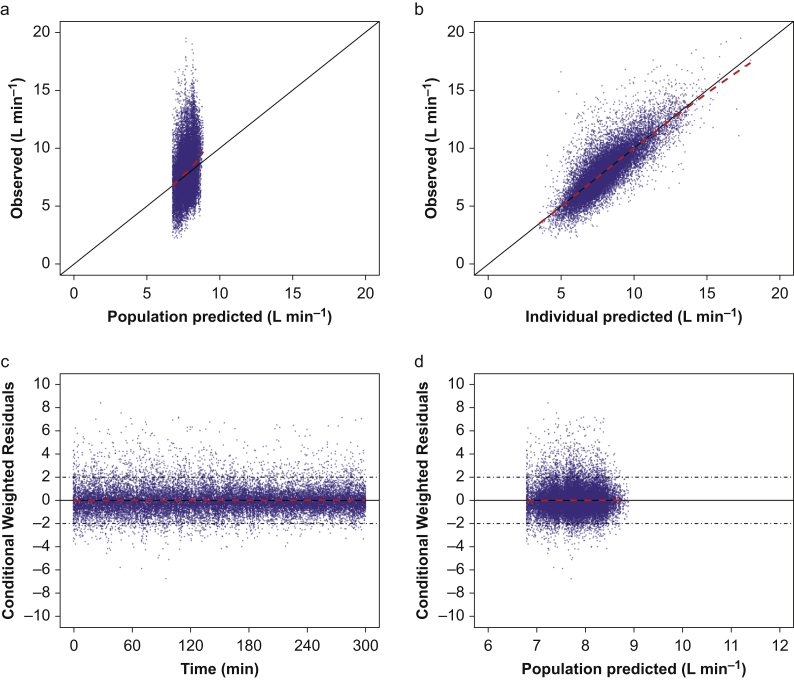
Goodness-of-fit plots. (a) Observed *vs* population predicted cardiac output, (b) observed *vs* individual predicted cardiac output, (c) conditional weighted residuals *vs* time, and (d) conditional weighted residuals *vs* population predicted cardiac output for Model 2. Red lines show locally weighted scatterplot smoothers to identify potential trends.

**Fig 4:**
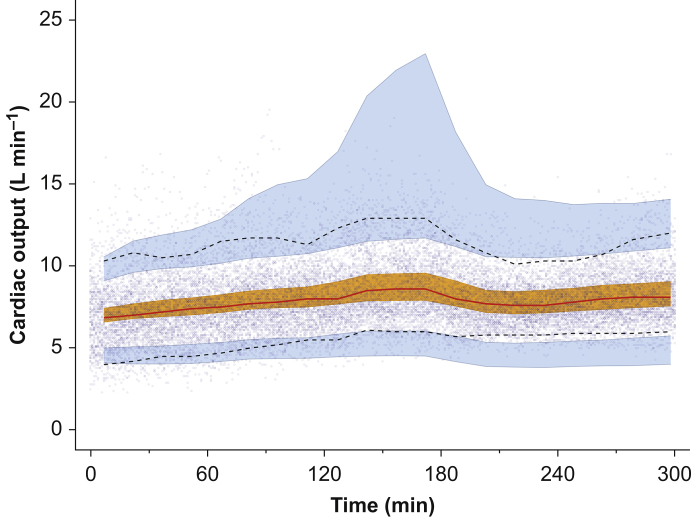
Visual predictive check based on the simulation of 1000 data sets from Model 2. The 50th, 5th, and 95th percentiles of the true data are shown by the red and lower and upper black lines, respectively. The orange and upper and lower blue shaded areas show the 95% confidence intervals of the simulated 50th (orange), 5th, and 95th (blue) percentile data. The dots are the measured cardiac-output data.

**Fig 5:**
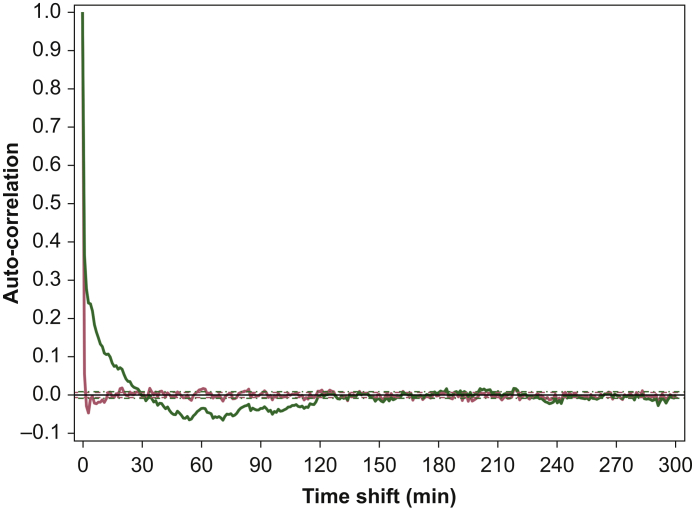
Auto-correlation function of the residuals of Model 2 (red line) and Model 5 (green line) for the total data set.

Goodness-of-fit plots and the visual predictive check for Model 2 are given in Fig 3, Fig 4. Auto-correlation function plots for Models 2 and 5 are shown in Fig. 5. The visual predictive check revealed a slight undershoot of the simulated fifth percentile data compared with that of the fifth percentile of the true data (lower black line and shaded area). Adding the Kalman filter improved the model fits and resulted in substantially improved goodness-of-fit plots, visual predictive checks (data not shown), and auto- and cross-validation values. This indicates that Model 2 has uncorrelated residuals and is to be preferred over Model 5. Finally, screening Model 2 for covariates failed to show significant effects of either ketamine administration form (e.g. *S*-ketamine *vs RS*-ketamine administration) or placebo *vs* SNP administration.

## Discussion

We observed a stereoselective effect of ketamine on cardiac output. Although *S*-ketamine increased cardiac output in a concentration-dependent manner, no effect of *R*-ketamine on cardiac output was detected in our data set. Additionally, we observed that, in contrast to *S*-ketamine, the active metabolite *S*-NK reduced cardiac output. There was no effect of the nitric oxide donor SNP on the effects of either *S*- or *RS*-ketamine.

Two earlier pharmacokinetic/pharmacodynamic studies on the effect of ketamine on cardiac output have been published. Sigtermans and colleagues^14^ administered increasing doses of *S*-ketamine to healthy volunteers and modelled the effect of *S*-ketamine and *S*-NK on cardiac output using a base model with trend term, but without controller or noise component. In that study, the increase in cardiac output after infusion of *S*-ketamine was well described by the *S*-ketamine concentration in plasma without any effect from *S*-NK. Olofsen and colleagues^12^ administered increasing *S*-ketamine pulsatile doses to healthy volunteers and patients diagnosed with chronic regional pain syndrome Type 1. They modelled the effect of just *S*-ketamine on cardiac output using a pharmacodynamic model with controller and noise component. In the current pharmacodynamic analyses, incorporation of a trend term and noise component (Kalman filter) contributed to the significant improvement of the description of the data (Model 2), whereas adding a controller did not; the negative contribution of NK allowed for the characterisation of the undershoot in the data.

The trend term described a slow change in effect over time, independent of the plasma ketamine concentration. Sigtermans and colleagues^14^ observed a positive trend term in their study on the effect of ketamine on anti-nociception. Possibly, the change in cardiac output of +0.38 L min^−1^ in 300 min in the current study may be related to the slow increase in concentration of DHNK and HNK. To confirm this hypothesis, we performed sequential metabolite effect testing of the base model without and with a trend term, but could not detect a significant contribution of either metabolite to the trend term. Other causes for the positive trend may be a slow increase in arterial carbon dioxide concentration attributable to the respiratory effects of ketamine, or anxiety related because of the psychedelic effects of ketamine.

In agreement with Olofsen and colleagues,^12^ we added a Kalman filter to the base model. The Kalman filter is a method to track the state of a system in the presence of random disturbances. These disturbances are to be distinguished from residual or measurement noise; here, they might affect physiological processes related to homeostasis, and because of the inertia of such processes, the disturbances lead to correlated residual noise in addition to the measurement noise. In the current study, auto-correlation (correlation between residuals) and cross-correlation (correlation between residuals and pharmacodynamic input) indicate absence of significant correlations in the model with a Kalman filter (Model 2), whereas the noise was correlated in the model without a Kalman filter (Model 5). This indicates a significant improvement in model performance with more reliable estimates of variability and deterministic model parameters. Additionally, data analyses without a Kalman filter yielded much larger ω^2^ values (data not shown). These findings agree with earlier studies exploring noisy respiratory data and transdermal opioid absorption.^15^^,^^16^

The absence of effect of *R*-ketamine on cardiac output agrees with earlier findings of a lesser potency of *R*-ketamine compared with *S*-ketamine on various endpoints. For example, Geisslinger and colleagues^17^ reported significant higher systolic and diastolic blood pressures after *S*-ketamine compared with *RS*-ketamine. Their results suggest that *S*-ketamine is mostly responsible for the observed cardiovascular effects associated with ketamine administration. Hence, *R*- and *S*-enantiomers differentially engage sympathetic activation, possibly related to differences in receptor activation. For example, *S*-ketamine is about twice as potent as *R*-ketamine in producing voltage and use-dependent blockade of the N-methyl-D-aspartate receptor.^18^ These data agree with observations that *S*-ketamine, at anaesthetic doses, is more potent in reducing the electroencephalogram power spectrum compared with anaesthetic doses of *R*- and *RS*-ketamine and the difference in analgesic potency between *S*- and *RS*-ketamine at sub-anaesthetic doses.^9^^,^^19^

Covariate analysis revealed absence of effects from the administration form (racemic ketamine or the *S*-isomer), or from absence or presence of the nitric oxide donor SNP. This later observation contrasts a study in rabbits that shows that L-arginine, a substrate of nitric oxide formation, attenuated ketamine-induced increase in renal sympathetic nerve activity.^8^ Possibly, the SNP dose in our study was too low to reduce cardiac output (in contrast to the effect of SNP on psychedelic symptoms). Additionally, compensatory mechanisms may have prevented any effect of low-dose SNP in our healthy and young population of volunteers.

Finally, we observed a negative contribution of *S*-NK on cardiac output, an effect that could explain the undershoot after ketamine infusion. In fact, *S*-NK counteracted the effect of *S*-ketamine on cardiac excitation. This finding agrees with an earlier modelling study, in which NK was anti-analgesic and counteracted the analgesic effects of ketamine.^20^ The mechanism of this antagonist effect remains unknown and may be related to a differential receptor activation profile of NK *vs* ketamine.^20^ However, as stated earlier, one needs to be rather careful in the interpretation of these findings from our complex modelling study.^20^ Additional proof from either animal or human studies is needed before any definitive conclusions regarding the effect of *S*-NK on cardiac output may be drawn.

In conclusion, we performed a pharmacodynamic modelling study that evaluated the effects of *R*- and *S*-ketamine and its most important metabolites on cardiac output in healthy male volunteers. Important findings were that, in contrast to *S*-ketamine, *R*-ketamine was devoid of effect on cardiac output, whereas *S*-NK counteracted the effect of *S*-ketamine by having a negative effect on cardiac output.

## Authors contributions

JK, data analysis, wrote the paper; MvV. project supervision and performed experiments; LA writing of paper, MN, project supervision and writing of paper, performed experiments; AD wrote the protocol, performed data analysis, and wrote the paper; EO data analysis and wrote the paper. All authors approved the final version of the paper.

## Declarations of interest

The authors declare that they have no conflicts of interest.
